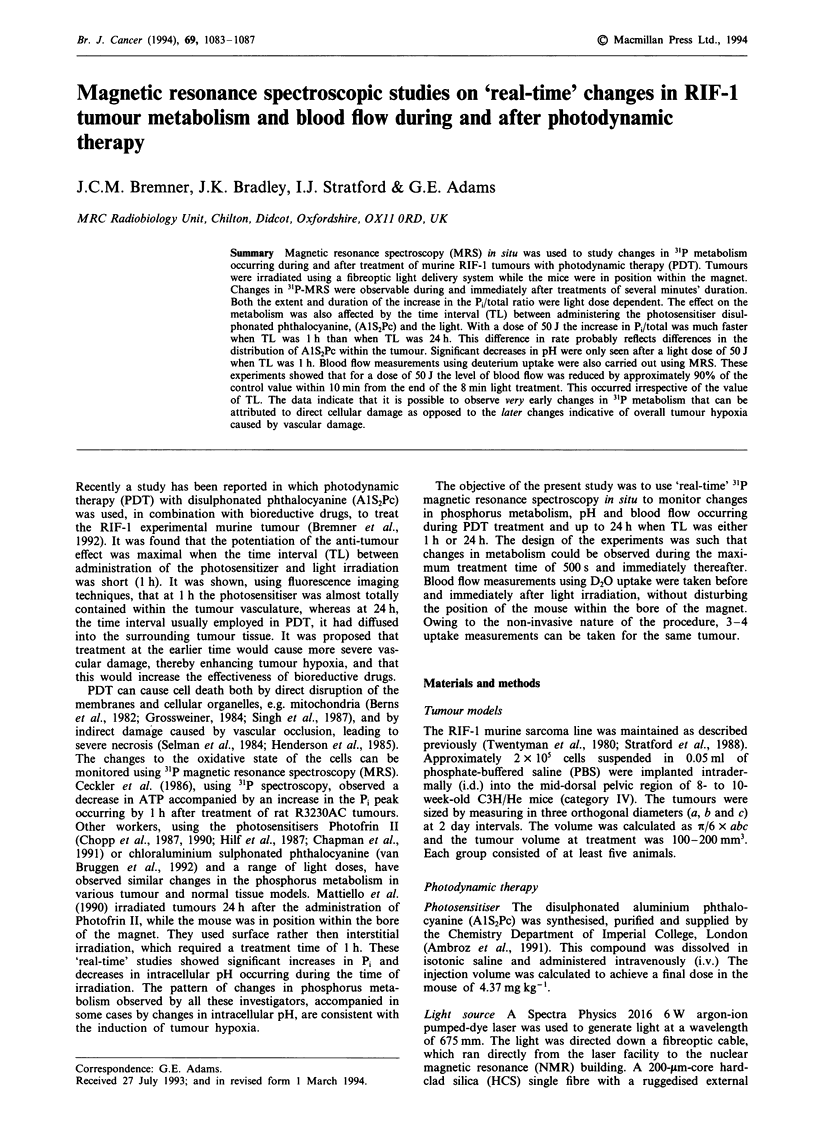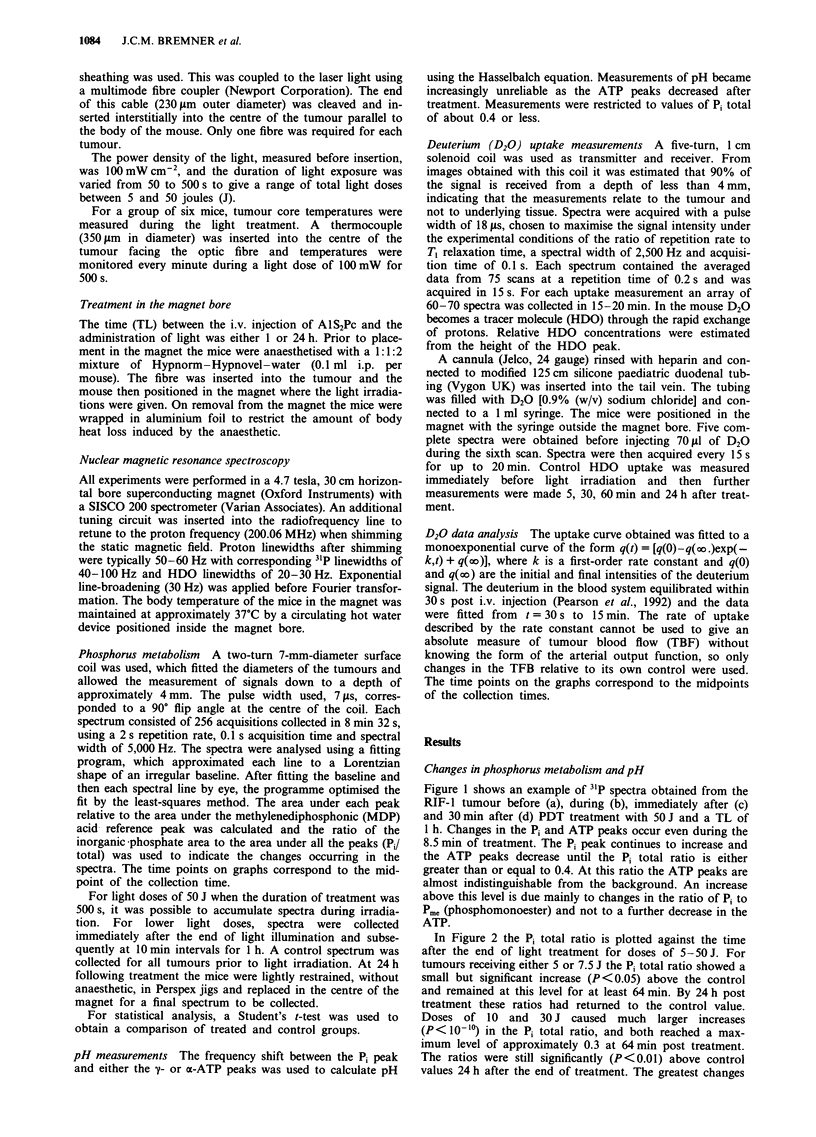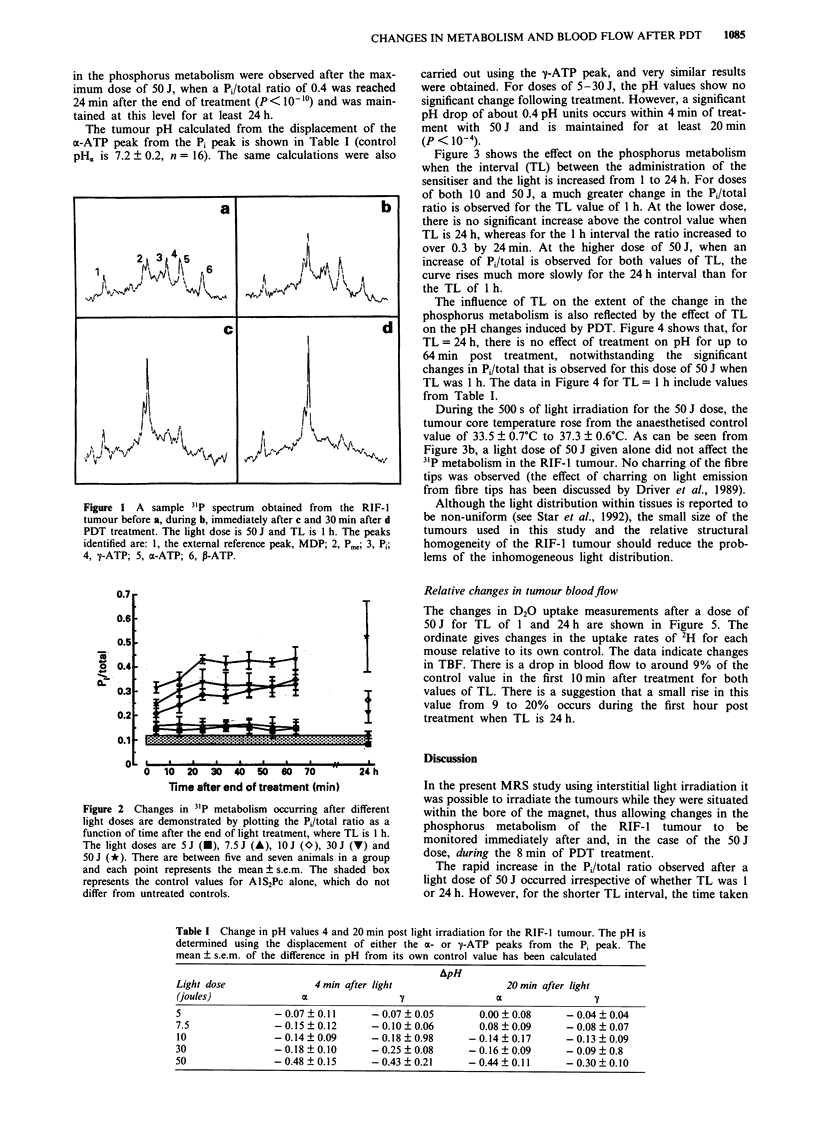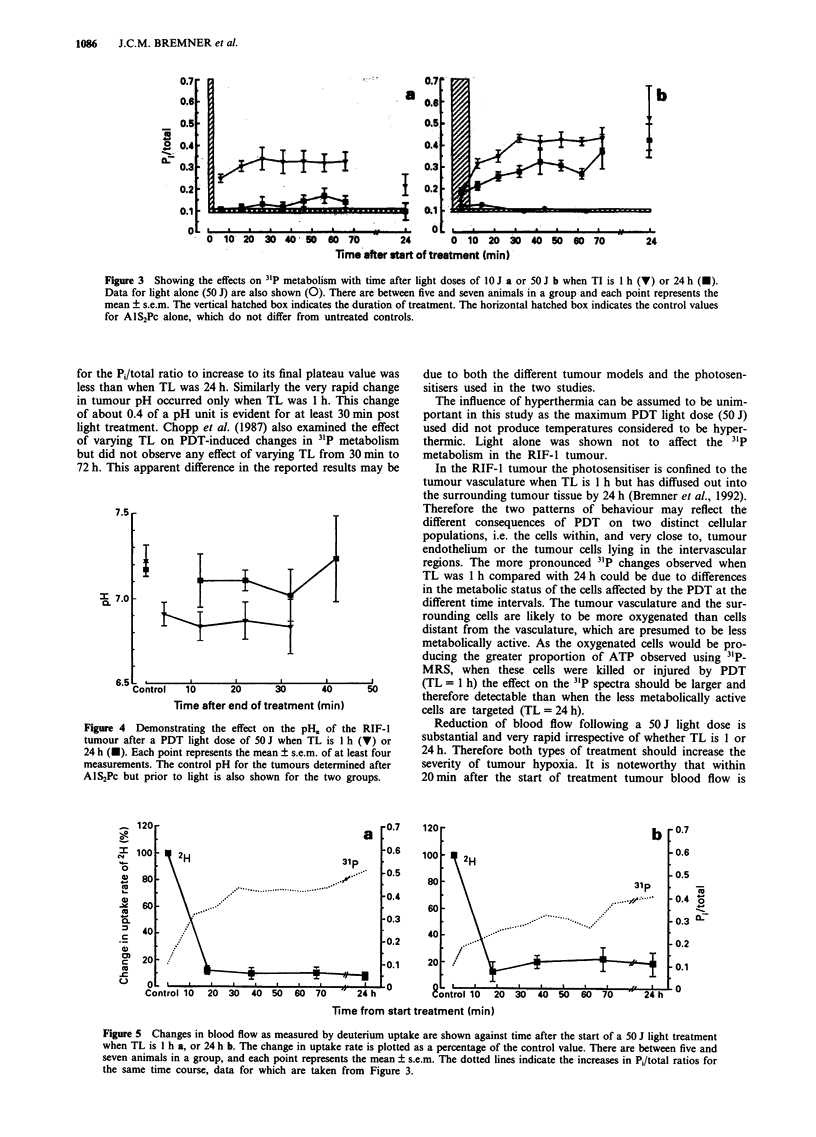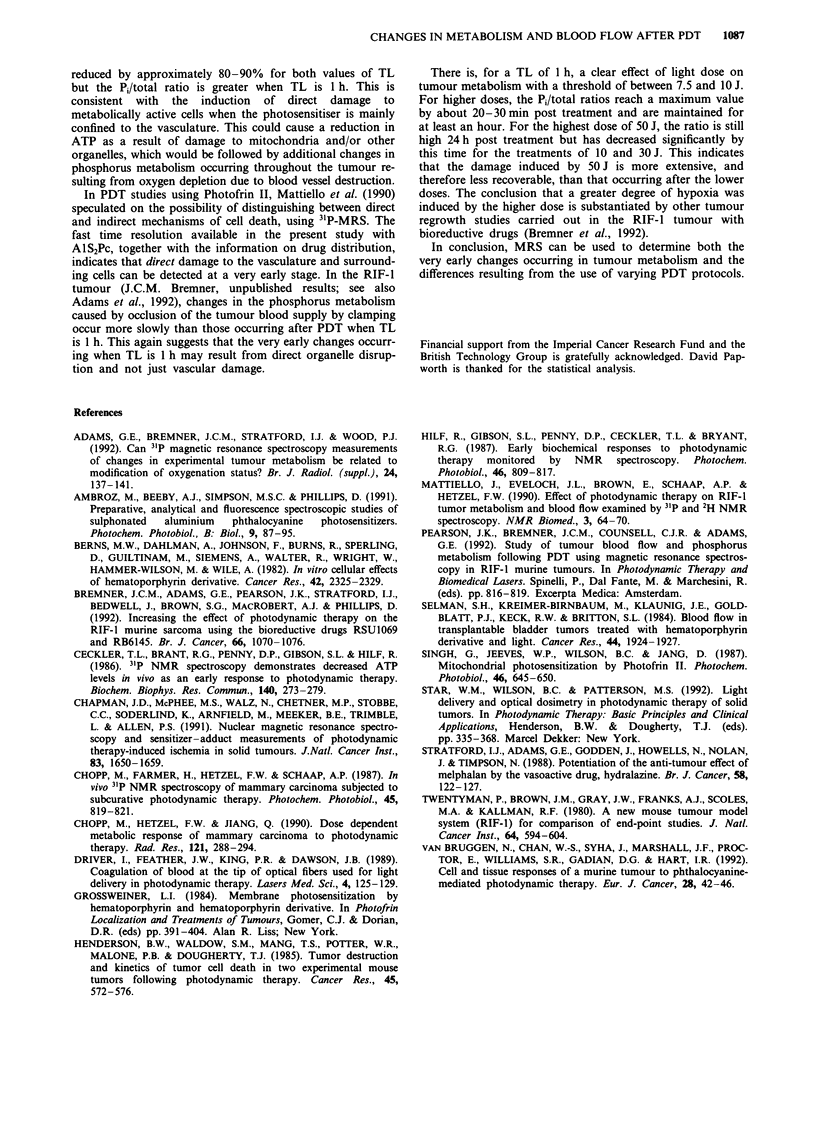# Magnetic resonance spectroscopic studies on 'real-time' changes in RIF-1 tumour metabolism and blood flow during and after photodynamic therapy.

**DOI:** 10.1038/bjc.1994.212

**Published:** 1994-06

**Authors:** J. C. Bremner, J. K. Bradley, I. J. Stratford, G. E. Adams

**Affiliations:** MRC Radiobiology Unit, Chilton, Didcot, Oxfordshire, UK.

## Abstract

Magnetic resonance spectroscopy (MRS) in situ was used to study changes in 31P metabolism occurring during and after treatment of murine RIF-1 tumours with photodynamic therapy (PDT). Tumours were irradiated using a fibreoptic light delivery system while the mice were in position within the magnet. Changes in 31P-MRS were observable during and immediately after treatments of several minutes' duration. Both the extent and duration of the increase in the Pi/total ratio were light dose dependent. The effect on the metabolism was also affected by the time interval (TL) between administering the photosensitiser disulphonated phthalocyanine, (A1S2Pc) and the light. With a dose of 50 J the increase in Pi/total was much faster when TL was 1 h than when TL was 24 h. This difference in rate probably reflects differences in the distribution of A1S2Pc within the tumour. Significant decreases in pH were only seen after a light dose of 50 J when TL was 1 h. Blood flow measurements using deuterium uptake were also carried out using MRS. These experiments showed that for a dose of 50 J the level of blood flow was reduced by approximately 90% of the control value within 10 min from the end of the 8 min light treatment. This occurred irrespective of the value of TL. The data indicate that it is possible to observe very early changes in 31P metabolism that can be attributed to direct cellular damage as opposed to the later changes indicative of overall tumour hypoxia caused by vascular damage.


Br. J. Cancer (1994), 69, 1083–1087 © Macmillan Press Ltd., 1994

### Magnetic resonance spectroscopic studies on 'real-time' changes in RIF-1 tumour metabolism and blood flow during and after photodynamic therapy

J.C.M. Bremner, J.K. Bradley, I.J. Stratford & G.E. Adams

*MRC Radiobiology Unit, Chilton, Didcot, Oxfordshire, OX11 0RD, UK*

**Summary** Magnetic resonance spectroscopy (MRS) *in situ* was used to study changes in $^{31}P$ metabolism occurring during and after treatment of murine RIF-1 tumours with photodynamic therapy (PDT). Tumours were irradiated using a fibreoptic light delivery system while the mice were in position within the magnet. Changes in $^{31}P$-MRS were observable during and immediately after treatments of several minutes' duration. Both the extent and duration of the increase in the $P_i$/total ratio were light dose dependent. The effect on the metabolism was also affected by the time interval (TL) between administering the photosensitiser disulphonated phthalocyanine, ($AlS_2Pc$) and the light. With a dose of 50 J the increase in $P_i$/total was much faster when TL was 1 h than when TL was 24 h. This difference in rate probably reflects differences in the distribution of $AlS_2Pc$ within the tumour. Significant decreases in pH were only seen after a light dose of 50 J when TL was 1 h. Blood flow measurements using deuterium uptake were also carried out using MRS. These experiments showed that for a dose of 50 J the level of blood flow was reduced by approximately 90% of the control value within 10 min from the end of the 8 min light treatment. This occurred irrespective of the value of TL. The data indicate that it is possible to observe *very* early changes in $^{31}P$ metabolism that can be attributed to direct cellular damage as opposed to the *later* changes indicative of overall tumour hypoxia caused by vascular damage.

Recently a study has been reported in which photodynamic therapy (PDT) with disulphonated phthalocyanine ($AlS_2Pc$) was used, in combination with bioreductive drugs, to treat the RIF-1 experimental murine tumour (Bremner *et al.*, 1992). It was found that the potentiation of the anti-tumour effect was maximal when the time interval (TL) between administration of the photosensitizer and light irradiation was short (1 h). It was shown, using fluorescence imaging techniques, that at 1 h the photosensitiser was almost totally contained within the tumour vasculature, whereas at 24 h, the time interval usually employed in PDT, it had diffused into the surrounding tumour tissue. It was proposed that treatment at the earlier time would cause more severe vascular damage, thereby enhancing tumour hypoxia, and that this would increase the effectiveness of bioreductive drugs.

PDT can cause cell death both by direct disruption of the membranes and cellular organelles, e.g. mitochondria (Berns *et al.*, 1982; Grossweiner, 1984; Singh *et al.*, 1987), and by indirect damage caused by vascular occlusion, leading to severe necrosis (Selman *et al.*, 1984; Henderson *et al.*, 1985). The changes to the oxidative state of the cells can be monitored using $^{31}P$ magnetic resonance spectroscopy (MRS). Ceckler *et al.* (1986), using $^{31}P$ spectroscopy, observed a decrease in ATP accompanied by an increase in the $P_i$ peak occurring by 1 h after treatment of rat R3230AC tumours. Other workers, using the photosensitisers Photofrin II (Chopp *et al.*, 1987, 1990; Hilf *et al.*, 1987; Chapman *et al.*, 1991) or chloraluminium sulphonated phthalocyanine (van Bruggen *et al.*, 1992) and a range of light doses, have observed similar changes in the phosphorus metabolism in various tumour and normal tissue models. Mattiello *et al.* (1990) irradiated tumours 24 h after the administration of Photofrin II, while the mouse was in position within the bore of the magnet. They used surface rather then interstitial irradiation, which required a treatment time of 1 h. These 'real-time' studies showed significant increases in $P_i$ and decreases in intracellular pH occurring during the time of irradiation. The pattern of changes in phosphorus metabolism observed by all these investigators, accompanied in some cases by changes in intracellular pH, are consistent with the induction of tumour hypoxia.

Correspondence: G.E. Adams.
Received 27 July 1993; and in revised form 1 March 1994.

The objective of the present study was to use 'real-time' $^{31}P$ magnetic resonance spectroscopy *in situ* to monitor changes in phosphorus metabolism, pH and blood flow occurring during PDT treatment and up to 24 h when TL was either 1 h or 24 h. The design of the experiments was such that changes in metabolism could be observed during the maximum treatment time of 500 s and immediately thereafter. Blood flow measurements using $D_2O$ uptake were taken before and immediately after light irradiation, without disturbing the position of the mouse within the bore of the magnet. Owing to the non-invasive nature of the procedure, 3–4 uptake measurements can be taken for the same tumour.

### Materials and methods

### *Tumour models*

The RIF-1 murine sarcoma line was maintained as described previously (Twentyman *et al.*, 1980; Stratford *et al.*, 1988). Approximately $2 \times 10^5$ cells suspended in 0.05 ml of phosphate-buffered saline (PBS) were implanted intradermally (i.d.) into the mid-dorsal pelvic region of 8- to 10-week-old C3H/He mice (category IV). The tumours were sized by measuring in three orthogonal diameters (*a*, *b* and *c*) at 2 day intervals. The volume was calculated as $\pi/6 \times abc$ and the tumour volume at treatment was 100–200 $mm^3$. Each group consisted of at least five animals.

### *Photodynamic therapy*

*Photosensitiser* The disulphonated aluminium phthalocyanine ($AlS_2Pc$) was synthesised, purified and supplied by the Chemistry Department of Imperial College, London (Ambroz *et al.*, 1991). This compound was dissolved in isotonic saline and administered intravenously (i.v.) The injection volume was calculated to achieve a final dose in the mouse of 4.37 mg $kg^{-1}$.

*Light source* A Spectra Physics 2016 6 W argon-ion pumped-dye laser was used to generate light at a wavelength of 675 mm. The light was directed down a fibreoptic cable, which ran directly from the laser facility to the nuclear magnetic resonance (NMR) building. A 200-μm-core hard-clad silica (HCS) single fibre with a ruggedised external sheathing was used. This was coupled to the laser light using a multimode fibre coupler (Newport Corporation). The end of this cable (230 μm outer diameter) was cleaved and inserted interstitially into the centre of the tumour parallel to the body of the mouse. Only one fibre was required for each tumour.

The power density of the light, measured before insertion, was 100 mW $cm^{-2}$, and the duration of light exposure was varied from 50 to 500 s to give a range of total light doses between 5 and 50 joules (J).

For a group of six mice, tumour core temperatures were measured during the light treatment. A thermocouple (350 μm in diameter) was inserted into the centre of the tumour facing the optic fibre and temperatures were monitored every minute during a light dose of 100 mW for 500 s.

*Treatment in the magnet bore*

The time (TL) between the i.v. injection of $AlS_2Pc$ and the administration of light was either 1 or 24 h. Prior to placement in the magnet the mice were anaesthetised with a 1:1:2 mixture of Hypnorm–Hypnovel–water (0.1 ml i.p. per mouse). The fibre was inserted into the tumour and the mouse then positioned in the magnet where the light irradiations were given. On removal from the magnet the mice were wrapped in aluminium foil to restrict the amount of body heat loss induced by the anaesthetic.

*Nuclear magnetic resonance spectroscopy*

All experiments were performed in a 4.7 tesla, 30 cm horizontal bore superconducting magnet (Oxford Instruments) with a SISCO 200 spectrometer (Varian Associates). An additional tuning circuit was inserted into the radiofrequency line to retune to the proton frequency (200.06 MHz) when shimming the static magnetic field. Proton linewidths after shimming were typically 50–60 Hz with corresponding $^{31}P$ linewidths of 40–100 Hz and HDO linewidths of 20–30 Hz. Exponential line-broadening (30 Hz) was applied before Fourier transformation. The body temperature of the mice in the magnet was maintained at approximately 37°C by a circulating hot water device positioned inside the magnet bore.

*Phosphorus metabolism* A two-turn 7-mm-diameter surface coil was used, which fitted the diameters of the tumours and allowed the measurement of signals down to a depth of approximately 4 mm. The pulse width used, 7 μs, corresponded to a 90° flip angle at the centre of the coil. Each spectrum consisted of 256 acquisitions collected in 8 min 32 s, using a 2 s repetition rate, 0.1 s acquisition time and spectral width of 5,000 Hz. The spectra were analysed using a fitting program, which approximated each line to a Lorentzian shape of an irregular baseline. After fitting the baseline and then each spectral line by eye, the programme optimised the fit by the least-squares method. The area under each peak relative to the area under the methylenediphosphonic (MDP) acid reference peak was calculated and the ratio of the inorganic phosphate area to the area under all the peaks ($P_i$/total) was used to indicate the changes occurring in the spectra. The time points on graphs correspond to the midpoint of the collection time.

For light doses of 50 J when the duration of treatment was 500 s, it was possible to accumulate spectra during irradiation. For lower light doses, spectra were collected immediately after the end of light illumination and subsequently at 10 min intervals for 1 h. A control spectrum was collected for all tumours prior to light irradiation. At 24 h following treatment the mice were lightly restrained, without anaesthetic, in Perspex jigs and replaced in the centre of the magnet for a final spectrum to be collected.

For statistical analysis, a Student's $t$-test was used to obtain a comparison of treated and control groups.

*pH measurements* The frequency shift between the $P_i$ peak and either the γ- or α-ATP peaks was used to calculate pH using the Hasselbalch equation. Measurements of pH became increasingly unreliable as the ATP peaks decreased after treatment. Measurements were restricted to values of $P_i$ total of about 0.4 or less.

*Deuterium ($D_2O$) uptake measurements* A five-turn, 1 cm solenoid coil was used as transmitter and receiver. From images obtained with this coil it was estimated that 90% of the signal is received from a depth of less than 4 mm, indicating that the measurements relate to the tumour and not to underlying tissue. Spectra were acquired with a pulse width of 18 μs, chosen to maximise the signal intensity under the experimental conditions of the ratio of repetition rate to $T_1$ relaxation time, a spectral width of 2,500 Hz and acquisition time of 0.1 s. Each spectrum contained the averaged data from 75 scans at a repetition time of 0.2 s and was acquired in 15 s. For each uptake measurement an array of 60–70 spectra was collected in 15–20 min. In the mouse $D_2O$ becomes a tracer molecule (HDO) through the rapid exchange of protons. Relative HDO concentrations were estimated from the height of the HDO peak.

A cannula (Jelco, 24 gauge) rinsed with heparin and connected to modified 125 cm silicone paediatric duodenal tubing (Vygon UK) was inserted into the tail vein. The tubing was filled with $D_2O$ [0.9% (w/v) sodium chloride] and connected to a 1 ml syringe. The mice were positioned in the magnet with the syringe outside the magnet bore. Five complete spectra were obtained before injecting 70 μl of $D_2O$ during the sixth scan. Spectra were then acquired every 15 s for up to 20 min. Control HDO uptake was measured immediately before light irradiation and then further measurements were made 5, 30, 60 min and 24 h after treatment.

*$D_2O$ data analysis* The uptake curve obtained was fitted to a monoexponential curve of the form $q(t) = [q(0) - q(\infty.)\exp(-k,t) + q(\infty)]$, where $k$ is a first-order rate constant and $q(0)$ and $q(\infty)$ are the initial and final intensities of the deuterium signal. The deuterium in the blood system equilibrated within 30 s post i.v. injection (Pearson *et al.*, 1992) and the data were fitted from $t = 30$ s to 15 min. The rate of uptake described by the rate constant cannot be used to give an absolute measure of tumour blood flow (TBF) without knowing the form of the arterial output function, so only changes in the TFB relative to its own control were used. The time points on the graphs correspond to the midpoints of the collection times.

### Results

*Changes in phosphorus metabolism and pH*

Figure 1 shows an example of $^{31}P$ spectra obtained from the RIF-1 tumour before (a), during (b), immediately after (c) and 30 min after (d) PDT treatment with 50 J and a TL of 1 h. Changes in the $P_i$ and ATP peaks occur even during the 8.5 min of treatment. The $P_i$ peak continues to increase and the ATP peaks decrease until the $P_i$ total ratio is either greater than or equal to 0.4. At this ratio the ATP peaks are almost indistinguishable from the background. An increase above this level is due mainly to changes in the ratio of $P_i$ to $P_{me}$ (phosphomonoester) and not to a further decrease in the ATP.

In Figure 2 the $P_i$ total ratio is plotted against the time after the end of light treatment for doses of 5–50 J. For tumours receiving either 5 or 7.5 J the $P_i$ total ratio showed a small but significant increase ($P < 0.05$) above the control and remained at this level for at least 64 min. By 24 h post treatment these ratios had returned to the control value. Doses of 10 and 30 J caused much larger increases ($P < 10^{-10}$) in the $P_i$ total ratio, and both reached a maximum level of approximately 0.3 at 64 min post treatment. The ratios were still significantly ($P < 0.01$) above control values 24 h after the end of treatment. The greatest changes in the phosphorus metabolism were observed after the maximum dose of 50 J, when a $P_i$/total ratio of 0.4 was reached 24 min after the end of treatment ($P < 10^{-10}$) and was maintained at this level for at least 24 h.

The tumour pH calculated from the displacement of the α-ATP peak from the $P_i$ peak is shown in Table I (control $pH_\alpha$ is 7.2 ± 0.2, $n = 16$). The same calculations were also carried out using the γ-ATP peak, and very similar results were obtained. For doses of 5–30 J, the pH values show no significant change following treatment. However, a significant pH drop of about 0.4 pH units occurs within 4 min of treatment with 50 J and is maintained for at least 20 min ($P < 10^{-4}$).

Figure 3 shows the effect on the phosphorus metabolism when the interval (TL) between the administration of the sensitiser and the light is increased from 1 to 24 h. For doses of both 10 and 50 J, a much greater change in the $P_i$/total ratio is observed for the TL value of 1 h. At the lower dose, there is no significant increase above the control value when TL is 24 h, whereas for the 1 h interval the ratio increased to over 0.3 by 24 min. At the higher dose of 50 J, when an increase of $P_i$/total is observed for both values of TL, the curve rises much more slowly for the 24 h interval than for the TL of 1 h.

The influence of TL on the extent of the change in the phosphorus metabolism is also reflected by the effect of TL on the pH changes induced by PDT. Figure 4 shows that, for TL = 24 h, there is no effect of treatment on pH for up to 64 min post treatment, notwithstanding the significant changes in $P_i$/total that is observed for this dose of 50 J when TL was 1 h. The data in Figure 4 for TL = 1 h include values from Table I.

During the 500 s of light irradiation for the 50 J dose, the tumour core temperature rose from the anaesthetised control value of 33.5 ± 0.7°C to 37.3 ± 0.6°C. As can be seen from Figure 3b, a light dose of 50 J given alone did not affect the $^{31}P$ metabolism in the RIF-1 tumour. No charring of the fibre tips was observed (the effect of charring on light emission from fibre tips has been discussed by Driver *et al.*, 1989).

Although the light distribution within tissues is reported to be non-uniform (see Star *et al.*, 1992), the small size of the tumours used in this study and the relative structural homogeneity of the RIF-1 tumour should reduce the problems of the inhomogeneous light distribution.

**Figure 1** A sample $^{31}P$ spectrum obtained from the RIF-1 tumour before **a**, during **b**, immediately after **c** and 30 min after **d** PDT treatment. The light dose is 50 J and TL is 1 h. The peaks identified are: 1, the external reference peak, MDP; 2, $P_{me}$; 3, $P_i$; 4, γ-ATP; 5, α-ATP; 6, β-ATP.

**Figure 2** Changes in $^{31}P$ metabolism occurring after different light doses are demonstrated by plotting the $P_i$/total ratio as a function of time after the end of light treatment, where TL is 1 h. The light doses are 5 J (■), 7.5 J (▲), 10 J (◇), 30 J (▼) and 50 J (★). There are between five and seven animals in a group and each point represents the mean ± s.e.m. The shaded box represents the control values for $AlS_2Pc$ alone, which do not differ from untreated controls.

*Relative changes in tumour blood flow*

The changes in $D_2O$ uptake measurements after a dose of 50 J for TL of 1 and 24 h are shown in Figure 5. The ordinate gives changes in the uptake rates of $^2H$ for each mouse relative to its own control. The data indicate changes in TBF. There is a drop in blood flow to around 9% of the control value in the first 10 min after treatment for both values of TL. There is a suggestion that a small rise in this value from 9 to 20% occurs during the first hour post treatment when TL is 24 h.

### Discussion

In the present MRS study using interstitial light irradiation it was possible to irradiate the tumours while they were situated within the bore of the magnet, thus allowing changes in the phosphorus metabolism of the RIF-1 tumour to be monitored immediately after and, in the case of the 50 J dose, *during* the 8 min of PDT treatment.

The rapid increase in the $P_i$/total ratio observed after a light dose of 50 J occurred irrespective of whether TL was 1 or 24 h. However, for the shorter TL interval, the time taken

**Table I** Change in pH values 4 and 20 min post light irradiation for the RIF-1 tumour. The pH is determined using the displacement of either the α- or γ-ATP peaks from the $P_i$ peak. The mean ± s.e.m. of the difference in pH from its own control value has been calculated

| | ΔpH | | | |
|---|---|---|---|---|
| *Light dose* | *4 min after light* | | *20 min after light* | |
| *(joules)* | α | γ | α | γ |
| 5 | − 0.07 ± 0.11 | − 0.07 ± 0.05 | 0.00 ± 0.08 | − 0.04 ± 0.04 |
| 7.5 | − 0.15 ± 0.12 | − 0.10 ± 0.06 | 0.08 ± 0.09 | − 0.08 ± 0.07 |
| 10 | − 0.14 ± 0.09 | − 0.18 ± 0.98 | − 0.14 ± 0.17 | − 0.13 ± 0.09 |
| 30 | − 0.18 ± 0.10 | − 0.25 ± 0.08 | − 0.16 ± 0.09 | − 0.09 ± 0.8 |
| 50 | − 0.48 ± 0.15 | − 0.43 ± 0.21 | − 0.44 ± 0.11 | − 0.30 ± 0.10 |

**Figure 3** Showing the effects on $^{31}P$ metabolism with time after light doses of 10 J **a** or 50 J **b** when TI is 1 h (▼) or 24 h (■). Data for light alone (50 J) are also shown (O). There are between five and seven animals in a group and each point represents the mean ± s.e.m. The vertical hatched box indicates the duration of treatment. The horizontal hatched box indicates the control values for $AlS_2Pc$ alone, which do not differ from untreated controls.

for the $P_i$/total ratio to increase to its final plateau value was less than when TL was 24 h. Similarly the very rapid change in tumour pH occurred only when TL was 1 h. This change of about 0.4 of a pH unit is evident for at least 30 min post light treatment. Chopp *et al.* (1987) also examined the effect of varying TL on PDT-induced changes in $^{31}P$ metabolism but did not observe any effect of varying TL from 30 min to 72 h. This apparent difference in the reported results may be

**Figure 4** Demonstrating the effect on the $pH_\alpha$ of the RIF-1 tumour after a PDT light dose of 50 J when TL is 1 h (▼) or 24 h (■). Each point represents the mean ± s.e.m. of at least four measurements. The control pH for the tumours determined after $AlS_2Pc$ but prior to light is also shown for the two groups.

due to both the different tumour models and the photosensitisers used in the two studies.

The influence of hyperthermia can be assumed to be unimportant in this study as the maximum PDT light dose (50 J) used did not produce temperatures considered to be hyperthermic. Light alone was shown not to affect the $^{31}P$ metabolism in the RIF-1 tumour.

In the RIF-1 tumour the photosensitiser is confined to the tumour vasculature when TL is 1 h but has diffused out into the surrounding tumour tissue by 24 h (Bremner *et al.*, 1992). Therefore the two patterns of behaviour may reflect the different consequences of PDT on two distinct cellular populations, i.e. the cells within, and very close to, tumour endothelium or the tumour cells lying in the intervascular regions. The more pronounced $^{31}P$ changes observed when TL was 1 h compared with 24 h could be due to differences in the metabolic status of the cells affected by the PDT at the different time intervals. The tumour vasculature and the surrounding cells are likely to be more oxygenated than cells distant from the vasculature, which are presumed to be less metabolically active. As the oxygenated cells would be producing the greater proportion of ATP observed using $^{31}P$-MRS, when these cells were killed or injured by PDT (TL = 1 h) the effect on the $^{31}P$ spectra should be larger and therefore detectable than when the less metabolically active cells are targeted (TL = 24 h).

Reduction of blood flow following a 50 J light dose is substantial and very rapid irrespective of whether TL is 1 or 24 h. Therefore both types of treatment should increase the severity of tumour hypoxia. It is noteworthy that within 20 min after the start of treatment tumour blood flow is

**Figure 5** Changes in blood flow as measured by deuterium uptake are shown against time after the start of a 50 J light treatment when TL is 1 h **a**, or 24 h **b**. The change in uptake rate is plotted as a percentage of the control value. There are between five and seven animals in a group, and each point represents the mean ± s.e.m. The dotted lines indicate the increases in $P_i$/total ratios for the same time course, data for which are taken from Figure 3.

reduced by approximately 80–90% for both values of TL but the $P_i$/total ratio is greater when TL is 1 h. This is consistent with the induction of direct damage to metabolically active cells when the photosensitiser is mainly confined to the vasculature. This could cause a reduction in ATP as a result of damage to mitochondria and/or other organelles, which would be followed by additional changes in phosphorus metabolism occurring throughout the tumour resulting from oxygen depletion due to blood vessel destruction.

In PDT studies using Photofrin II, Mattiello *et al.* (1990) speculated on the possibility of distinguishing between direct and indirect mechanisms of cell death, using $^{31}$P-MRS. The fast time resolution available in the present study with $AlS_2Pc$, together with the information on drug distribution, indicates that *direct* damage to the vasculature and surrounding cells can be detected at a very early stage. In the RIF-1 tumour (J.C.M. Bremner, unpublished results; see also Adams *et al.*, 1992), changes in the phosphorus metabolism caused by occlusion of the tumour blood supply by clamping occur more slowly than those occurring after PDT when TL is 1 h. This again suggests that the very early changes occurring when TL is 1 h may result from direct organelle disruption and not just vascular damage.

There is, for a TL of 1 h, a clear effect of light dose on tumour metabolism with a threshold of between 7.5 and 10 J. For higher doses, the $P_i$/total ratios reach a maximum value by about 20–30 min post treatment and are maintained for at least an hour. For the highest dose of 50 J, the ratio is still high 24 h post treatment but has decreased significantly by this time for the treatments of 10 and 30 J. This indicates that the damage induced by 50 J is more extensive, and therefore less recoverable, than that occurring after the lower doses. The conclusion that a greater degree of hypoxia was induced by the higher dose is substantiated by other tumour regrowth studies carried out in the RIF-1 tumour with bioreductive drugs (Bremner *et al.*, 1992).

In conclusion, MRS can be used to determine both the very early changes occurring in tumour metabolism and the differences resulting from the use of varying PDT protocols.

Financial support from the Imperial Cancer Research Fund and the British Technology Group is gratefully acknowledged. David Papworth is thanked for the statistical analysis.

### References

ADAMS, G.E., BREMNER, J.C.M., STRATFORD, I.J. & WOOD, P.J. (1992). Can $^{31}$P magnetic resonance spectroscopy measurements of changes in experimental tumour metabolism be related to modification of oxygenation status? *Br. J. Radiol. (suppl.)*, **24**, 137–141.

AMBROZ, M., BEEBY, A.J., SIMPSON, M.S.C. & PHILLIPS, D. (1991). Preparative, analytical and fluorescence spectroscopic studies of sulphonated aluminium phthalocyanine photosensitizers. *Photochem. Photobiol., B: Biol.*, **9**, 87–95.

BERNS, M.W., DAHLMAN, A., JOHNSON, F., BURNS, R., SPERLING, D., GUILTINAM, M., SIEMENS, A., WALTER, R., WRIGHT, W., HAMMER-WILSON, M. & WILE, A. (1982). *In vitro* cellular effects of hematoporphyrin derivative. *Cancer Res.*, **42**, 2325–2329.

BREMNER, J.C.M., ADAMS, G.E., PEARSON, J.K., STRATFORD, I.J., BEDWELL, J., BROWN, S.G., MACROBERT, A.J. & PHILLIPS, D. (1992). Increasing the effect of photodynamic therapy on the RIF-1 murine sarcoma using the bioreductive drugs RSU1069 and RB6145. *Br. J. Cancer*, **66**, 1070–1076.

CECKLER, T.L., BRANT, R.G., PENNY, D.P., GIBSON, S.L. & HILF, R. (1986). $^{31}$P NMR spectroscopy demonstrates decreased ATP levels *in vivo* as an early response to photodynamic therapy. *Biochem. Biophys. Res. Commun.*, **140**, 273–279.

CHAPMAN, J.D., McPHEE, M.S., WALZ, N., CHETNER, M.P., STOBBE, C.C., SODERLIND, K., ARNFIELD, M., MEEKER, B.E., TRIMBLE, L. & ALLEN, P.S. (1991). Nuclear magnetic resonance spectroscopy and sensitizer-adduct measurements of photodynamic therapy-induced ischemia in solid tumours. *J.Natl. Cancer Inst.*, **83**, 1650–1659.

CHOPP, M., FARMER, H., HETZEL, F.W. & SCHAAP, A.P. (1987). *In vivo* $^{31}$P NMR spectroscopy of mammary carcinoma subjected to subcurative photodynamic therapy. *Photochem. Photobiol.*, **45**, 819–821.

CHOPP, M., HETZEL, F.W. & JIANG, Q. (1990). Dose dependent metabolic response of mammary carcinoma to photodynamic therapy. *Rad. Res.*, **121**, 288–294.

DRIVER, I., FEATHER, J.W., KING, P.R. & DAWSON, J.B. (1989). Coagulation of blood at the tip of optical fibers used for light delivery in photodynamic therapy. *Lasers Med. Sci.*, **4**, 125–129.

GROSSWEINER, L.I. (1984). Membrane photosensitization by hematoporphyrin and hematoporphyrin derivative. In *Photofrin Localization and Treatments of Tumours*, Gomer, C.J. & Dorian, D.R. (eds) pp. 391–404. Alan R. Liss; New York.

HENDERSON, B.W., WALDOW, S.M., MANG, T.S., POTTER, W.R., MALONE, P.B. & DOUGHERTY, T.J. (1985). Tumor destruction and kinetics of tumor cell death in two experimental mouse tumors following photodynamic therapy. *Cancer Res.*, **45**, 572–576.

HILF, R., GIBSON, S.L., PENNY, D.P., CECKLER, T.L. & BRYANT, R.G. (1987). Early biochemical responses to photodynamic therapy monitored by NMR spectroscopy. *Photochem. Photobiol.*, **46**, 809–817.

MATTIELLO, J., EVELOCH, J.L., BROWN, E., SCHAAP, A.P. & HETZEL, F.W. (1990). Effect of photodynamic therapy on RIF-1 tumor metabolism and blood flow examined by $^{31}$P and $^{2}$H NMR spectroscopy. *NMR Biomed.*, **3**, 64–70.

PEARSON, J.K., BREMNER, J.C.M., COUNSELL, C.J.R. & ADAMS, G.E. (1992). Study of tumour blood flow and phosphorus metabolism following PDT using magnetic resonance spectroscopy in RIF-1 murine tumours. In *Photodynamic Therapy and Biomedical Lasers*. Spinelli, P., Dal Fante, M. & Marchesini, R. (eds). pp. 816–819. Excerpta Medica: Amsterdam.

SELMAN, S.H., KREIMER-BIRNBAUM, M., KLAUNIG, J.E., GOLDBLATT, P.J., KECK, R.W. & BRITTON, S.L. (1984). Blood flow in transplantable bladder tumors treated with hematoporphyrin derivative and light. *Cancer Res.*, **44**, 1924–1927.

SINGH, G., JEEVES, W.P., WILSON, B.C. & JANG, D. (1987). Mitochondrial photosensitization by Photofrin II. *Photochem. Photobiol.*, **46**, 645–650.

STAR, W.M., WILSON, B.C. & PATTERSON, M.S. (1992). Light delivery and optical dosimetry in photodynamic therapy of solid tumors. In *Photodynamic Therapy: Basic Principles and Clinical Applications*, Henderson, B.W. & Dougherty, T.J. (eds). pp. 335–368. Marcel Dekker: New York.

STRATFORD, I.J., ADAMS, G.E., GODDEN, J., HOWELLS, N., NOLAN, J. & TIMPSON, N. (1988). Potentiation of the anti-tumour effect of melphalan by the vasoactive drug, hydralazine. *Br. J. Cancer*, **58**, 122–127.

TWENTYMAN, P., BROWN, J.M., GRAY, J.W., FRANKS, A.J., SCOLES, M.A. & KALLMAN, R.F. (1980). A new mouse tumour model system (RIF-1) for comparison of end-point studies. *J. Natl. Cancer Inst.*, **64**, 594–604.

VAN BRUGGEN, N., CHAN, W.-S., SYHA, J., MARSHALL, J.F., PROCTOR, E., WILLIAMS, S.R., GADIAN, D.G. & HART, I.R. (1992). Cell and tissue responses of a murine tumour to phthalocyanine-mediated photodynamic therapy. *Eur. J. Cancer*, **28**, 42–46.